# Editorial: Advancing Science for Clinical Care in MDS

**DOI:** 10.3389/fonc.2022.901118

**Published:** 2022-04-21

**Authors:** Maria E. Figueroa, Amy E. DeZern

**Affiliations:** ^1^ Sidney Kimmel Comprehensive Cancer Center, Johns Hopkins Medicine, Baltimore, MD, United States; ^2^ Sylvester Comprehensive Cancer Center, University of Miami Health System, Miami, FL, United States

**Keywords:** clonal hematopoiesis, BMT, MDS, mouse model, IBMFS

## Introduction

The myelodysplastic syndromes (MDS) are collectively the most common myeloid neoplasms. Clonal hematopoiesis (CH) present in these diseases results in bone marrow failure characteristically seen in patients and likely facilitates potential development of acute myeloid leukemia. The heterogeneity of MDS pathobiology and lack of adequate laboratory models has historically posed a challenge to complete biologic understanding as well as the development of newer therapies, including improvements in the only path to cure for MDS with bone marrow transplantation (BMT).

In the laboratory, research in MDS has been hindered by the lack of preclinical models that faithfully replicate the complexity of the disease and capture its heterogeneity. The complex molecular landscape of the disease poses a unique challenge when creating transgenic mouse-models that accurately recapitulate all features of the disease. In this review, Liu et al. provide a comprehensive overview of the currently available murine models and comment on applicability in MDS specifically. In addition, the pro-apoptotic nature of primary MDS cells makes their recovery after biobanking extremely challenging, with very low viability percentages being routine when thawing primary specimens. This, along with the fact that these primary MDS cells are difficult to culture and manipulate ex vivo, have historically limited *in vitro* studies and resulted in a paucity of cell lines and patient-derived xenograft models. In recent years, a better understanding of the mutational landscape of MDS along with the development of novel immunocompromised murine models have paved the way for the development of improved transgenic and xenograft murine models. Researchers and clinicians alike will benefit from this series to enhance knowledge of these topics.

In addition to the technical challenges posed by the lack of faithful *in vivo* and *in vitro* models, basic and translational research in MDS has also been hindered by lack of proper understanding of the complexity of the disease. Far from being a single disease, MDS constitutes a heterogeneous group of clonal hematopoietic stem cell disorders, with some shared features but also distinct characteristics. Moreover, even within each of the currently recognized subtypes of MDS according to the World Health Organization, the malignant cells in the bone marrow are not homogeneous, and while a malignant stem cell has been empirically demonstrated to originate the disease, other malignant progenitors in the bone marrow as well as immune and stromal cells have been shown to play contributing roles to MDS pathogenesis.

Zhang et al. describe in detail how the development of single-cell sequencing techniques in recent years have transformed our understanding of normal hematopoiesis. As these techniques have improved, they can now be applied in the lab to describe the clonal heterogeneity in MDS, providing a comprehensive landscape of MDS subpopulations in the bone marrow as well as a progressive description of disease evolution. As experts in the field, they reveal a future role for the use of these methods in research and possibly clinical care.

At the bedside, aberrant clonal hematopoiesis is a known risk factor for the development of MDS. Chan et al. explain the role and relevance of this issue in their review. The development and cellular fitness of CH is shaped by aging, environmental exposures, and the germline (inherited) genetic background of an individual. However, the precise mechanisms that underlie progression from CH to MDS and the factors that put certain patients at higher risk for this progression are not fully understood. Novel directions and areas of investigation are already in place, leveraging the salient mouse models to inform future therapeutic approaches to CH and MDS. As research into these areas advances, MDS patients will likely increasingly benefit from the insights gained into the implications of CH for clinical outcome. Additionally, the distinction between somatic and inherited CH is critical as the confirmation of a suspected germline predisposition informs therapeutic considerations, guides monitoring pre- and post-treatment, and allows for genetic counseling. Studies of MDS with germline predisposition have provided unique insights into the pathogenesis and mechanisms of somatic genetic rescue that are likely applicable to acquired disease as well. Avagyan and Shimamura review the current data from the pediatric MDS field specifically as it relates to inherited predisposition and draw parallels for somatic disease.

Lastly, allogeneic BMT is the only treatment that can offer cure for MDS due to its potential to correct the aberrant CH in both somatic and inherited disease. However, concerns about patient suitability and approach to transplant in this disease remain, given mediocre outcomes in general for MDS patients post BMT. Jain and Elmariah review appropriate methodology to improve on long-term disease free remissions and mitigate toxicity from the procedure. Clinical review of candidacy, timing of transplant, and outcomes of these patients allows for additional research questions at all levels.

Ultimately, the advances described in this topic at the bench and at the bedside will allow improved diagnostic accuracy, provide insights into pathogenesis and molecular characterization of MDS on the path to refining therapeutic options for all MDS patients.

## Author Contributions

MF and AD contributed equally to this editorial and approved the submitted version.

## Conflict of Interest

The authors declare that the research was conducted in the absence of any commercial or financial relationships that could be construed as a potential conflict of interest.

## Publisher’s Note

All claims expressed in this article are solely those of the authors and do not necessarily represent those of their affiliated organizations, or those of the publisher, the editors and the reviewers. Any product that may be evaluated in this article, or claim that may be made by its manufacturer, is not guaranteed or endorsed by the publisher.

